# The hidden impact of a healthy-worker effect on the results of the Diesel Exhaust in Miners Study

**DOI:** 10.1007/s10654-016-0161-7

**Published:** 2016-05-25

**Authors:** Matthias Möhner

**Affiliations:** Division of Work and Health, Federal Institute for Occupational Safety and Health, Nöldnerstr. 40/42, 10317 Berlin, Germany

The Diesel Exhaust in Miners Study (DEMS) provides the most suitable epidemiological data on the association between diesel motor exhaust (DME) and lung cancer risk. The study base comprises underground and surface workers whose exposure to respirable elemental carbon (REC) differs by nearly two orders of magnitude. The data have been analysed using a cohort approach as well as a nested case–control approach. The primary cohort analyses revealed no association between DME and lung cancer [1]. However, adjusting for work location (“ever-underground” vs. “surface-only”) resulted in a dose–response relationship both in the cohort and the case–control analyses [1, 2].

Based mainly on the findings of the DEMS, a working group of the International Agency for Research on Cancer classified DME 2012 as “carcinogenic to humans” [3]. Subsequently, the disparity between DEMS results led to several critical commentaries. Moreover, the results are quite different from those of the German potash miners’ cohort study, published here in the journal [4]. Recently, an expert panel, set up by the Health Effects Institute, has evaluated the DEMS results [5] but did not provide an explanation for the apparently self-contradictory DEMS results.

For these reasons, it is worth taking a closer look at the results of the DEMS to scrutinize the disparate findings. The standardized mortality ratio (SMR) for lung cancer was slightly higher in surface-only workers than in ever-underground workers (1.33 vs. 1.21) [1]. However, the distribution of controls in the cases-control approach clearly shows that there were significantly more never-smokers (34 vs. 22 %) and significantly less heavy smokers (6 vs. 14 %) among surface-only workers than among ever-underground workers [2]. Moreover, mortality due to pneumoconiosis was considerably higher in ever-underground workers (SMR = 16.21 vs. 6.13) [1], indicating that underground workers are at higher risk due to former dust exposures. It is questionable whether these findings are compatible with the remarkable exposure–response relationship between REC exposure and lung cancer risk, especially in light of the huge differences in REC intensity according to work location.

The initial internal analysis of the complete cohort did not reveal any indication of harmful effects of REC on lung cancer risk. The exposure–response relationship only became clearly positive once work location was taken into account. And thus investigators also considered this variable in their case–control approach. Another critical aspect is that all REC related risk estimates were unusually adjusted by cross-product variables of the two baseline smoking variables (smoking status and smoking intensity) with work location. This led to a total of 19 parameters which had to be estimated for adjustment purposes only. This procedure seems inefficient and inappropriate in view of the mere 198 lung cancer cases.

As the DEMS cohort and case–control analyses offer evidence for a strong impact of the variable “work location” on the risk estimators, the content-related meaning of this factor is of special interest. In fact, in underground mines a regular medical check-up, including a screening for pneumoconiosis, is generally performed for all miners. In case of an atypical chest X-ray, which indicates early pneumoconiosis, the miner is moved from his underground job to a low-dust workplace at surface. Hence, a move from underground to surface work is generally determined by deteriorating health. On the contrary, an excellent physical condition is a prerequisite for the change of work location in the opposite direction. Usually, a positive pre-(underground)employment medical check-up is required in such a situation. Therefore, from a methodological point of view, the change of work location from surface to underground leads to a healthy-worker effect (HWE).

At first glance, it might appear unrealistic to consider a HWE in a miner cohort of prevalent hires. Yet, the cross-tabulation of various subgroups of miners and lung cancer deaths shows that the lung cancer death rate among surface-only and underground-only workers was about twice that of the remaining workers [6]. Combining all available data, the cohort can be divided into four categories: underground-only (33 %), surface-only (32 %), surface-first (15 %), and other workers (20 %). Therefore, it seems quite likely that the impact of HWE can be observed in surface-first workers.

The DEMS findings provide support data for this HWE-hypothesis. The risk estimates increase with increasing average REC intensity for individuals who ever held underground jobs [2]. Given the huge difference between underground and surface jobs in terms of REC exposure, the average REC intensity approximately reflects the share of surface work in the overall exposure duration. Hence, the surface-first workers are overrepresented in the first quartile, which acts as the reference category. In turn, one can assume that the lung cancer risk in this reference category is much lower than in a corresponding population group due to the HWE. In contrast, underground-only miners form the upper quartile and their lung cancer risk can be assumed to be higher than in the reference category even in absence of an exposure–response relationship between REC and lung cancer.

The hypothesis of a strong HWE among the surface-first workers may also explain the slightly higher SMR among surface-only workers in comparison to ever-underground workers: only those workers switched to underground, who fulfilled the strong medical requirements for underground work, and, hence, these workers improved the average health status of the ever-underground group.

In summary, there are some hints which point towards a bias in the DEMS results with respect to a HWE. From a methodological point of view the breakdown of the cohort into surface-only and ever-underground sub-cohorts seems incorrect. Excluding the results based on the adjustment for work location, the DEMS analyses do not show any noticeable risk increase with increasing REC exposure [1], a finding that has also been derived from the German potash miner cohort study [4]. Moreover, an approximation by unconditional logistic regression, based on the case–control data [2, Table 2], yields an odds ratio of 0.92 (95 % CI 0.66–1.30) for miners who ever worked underground, adjusted for smoking.

In conclusion, the evidence for an exposure–response relationship between DME and lung cancer is potentially much weaker than assumed by the DEMS investigators. A complete reanalysis of the DEMS data is recommended.
